# Setting up a Local ADHD service - reflections and pitfalls

**DOI:** 10.1192/j.eurpsy.2026.11174

**Published:** 2026-07-31

**Authors:** D. Collins, L. Riches, J. Beezhold

**Affiliations:** 1 Youth community, NSFT, Great Yarmouth; 2 Adult ADHD, NSFT, Norwich; 3 GYAS, NSFT, Great Yarmouth, United Kingdom

## Abstract

**Introduction:**

In October 2023, we set up a new,local ADHD servcie for adolescentsa age 18-25 years old This was in response to local need and because wait times for assesment in Adult service was excessive (4 years +) The aim of this new service was to screen, assess (when needed) and start treatment to young people in this geographcial area to try to prevent further disruption to educaiton, family and social life.

**Objectives:**

To accuratly assess, diagnose and manage ADHD in this population group.

**Methods:**

1. set up a new, monthly clinic specifically to assess ADHD
2. ADHD screeenrs sent out, only if clinical impression was that ADHD migth be a possibility
3. Booked in for 45 minute pre assesment session
4. Collateral history
5. Full ADHD assesment, if indicated, using DIVA 5

**Results:**

23 young people offered pre assessments 21 seen 11 booked for a full ADHD assessment 9 diagnosed with ADHD 4 referred on to Adult ADHD Of those diagnosd with ADHD, 8 opted for medicaiton. This was started, reviewed and monitored.

**Conclusions:**

Despite being more work than initally anticipated, this local servcie has been a success. over 60% of the young people seen were assessed as not havign ADHD, so we are certainly not overdiagnosing. the ones who did have ADHD responded well to medicaiton (many stopped other psychotropics, as these were no longer needed) outcomes were positive signficant reduction in morbidity and presure on Adutl ADHD servcie locally.

**Disclosure of Interest:**

None Declared

